# Sinonasal Renal Cell-Like Adenocarcinoma

**DOI:** 10.7759/cureus.14285

**Published:** 2021-04-04

**Authors:** Ellen L Tokarz, Nicole M Favre, William J Belles

**Affiliations:** 1 Otolaryngology - Head and Neck Surgery, Jacobs School of Medicine and Biomedical Sciences, University at Buffalo, Buffalo, USA; 2 Otolaryngology, Jacobs School of Medicine and Biomedical Sciences, University at Buffalo, Buffalo, USA

**Keywords:** nasal cavity, sinonasal tumor, skull base tumors, head and neck neoplasms, rhinology

## Abstract

Sinonasal renal cell-like adenocarcinoma (SNRCLA) is a newly defined, rare malignant tumor of the nasal cavity. The clinical course and response to treatment remain uncertain. The purpose of this study is to report a new case of SNRCLA and review the literature to determine clinical characteristics, treatment options, and outcomes. A 26-year-old male presented with headache, epistaxis, and nasal obstruction. Physical examination revealed a tumor involving bilateral ethmoid sinuses and MRI revealed extension through the cribriform plate. Surgical excision with endonasal and a bifrontal craniotomy was performed followed by adjuvant radiotherapy (RT). After RT, the patient had persistent disease requiring salvage surgery.

There are few previously reported cases of SNRCLA. A literature review yielded 14 previously reported cases with convincing diagnostic evidence of SNRCLA. Common presenting symptoms were epistaxis and nasal obstruction. Surgical excision was the primary treatment in fourteen cases, nine received RT, and none received chemotherapy. However, three cases had persistent or recurrent disease. Surgical excision is the mainstay of treatment for SNRCLA and adjuvant RT has been used in some patients with varying outcomes. The tumor is low grade with no reported cases of metastases or death. The best practice for treatment is yet to be determined.

## Introduction

Malignancies of the sinonasal tract are rare, accounting for only 3% of head and neck carcinomas and 0.5% of all malignancies [1]. Sinonasal renal cell-like adenocarcinoma (SNRCLA) is an extremely rare tumor, with the first two cases reported in 2002 by Zur et al. and Moh’d Hadi et al. [2,3]. The microscopic description of SNRCLA has been discussed in recent literature, providing evidence of a varied histological appearance with positive expression for markers such as cytokeratin 7 (CK7), S100, and vimentin. The histological resemblance of SNRCLA to renal cell carcinoma (RCC) has been consistently noted. Both tumors have translucent cytoplasm with cuboidal to columnar shape and distinct cytoplasmic borders. Therefore, renal imaging must be completed to rule out RCC when diagnosing SNRCLA [4]. Due to the rarity of this tumor, the standard of treatment has not yet been determined.

Although several studies report the pathologic characteristics of SNRCLA, the clinical course and response to treatment is not well described in the current literature. We report a new case of SNRCLA and review previously reported cases to determine its clinical characteristics and outcomes.

## Case presentation

**Figure 1:**
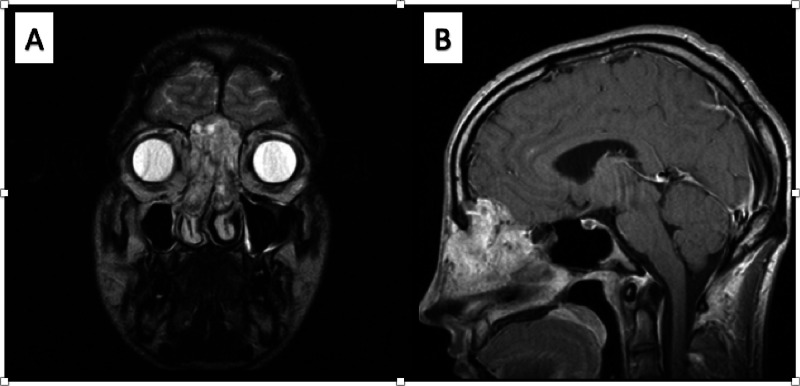
MRI imaging (A) Coronal MRI and (B) sagittal MRI revealing a 5.1 cm × 3.3 cm × 3.4 cm enhancing soft tissue mass centered in the ethmoid sinuses with invasion through the ethmoid plate and abutment of the bilateral frontal lobes. No gross invasion of frontal lobe parenchyma or dura identified.

A 26-year-old male presented to our institution with three to four months of epistaxis, bilateral nasal obstruction, hyposmia, and headaches. Physical examination revealed a large bilateral intranasal mass emanating from the superior nasal vault and extending into bilateral ethmoid sinuses. Magnetic resonance imaging (MRI) with gadolinium contrast revealed a 5.1 cm × 3.3 cm × 3.4 cm enhancing soft tissue mass centered in the ethmoid sinuses with invasion through the ethmoid plate abutting bilateral frontal lobes (Figure 1). There was no gross invasion of frontal lobe parenchyma or dura. Primary surgical excision was performed by otolaryngology and neurosurgical teams with a combined endonasal and bifrontal craniotomy approach. The patient underwent bilateral endoscopic ethmoidectomy and frontal sinusotomy, frontal sinus obliteration, and cranialization via coronal approach with pericranial flap. Intraoperatively, there was extensive bleeding and the patient required intraoperative transfusion of four units packed red blood cells. All gross disease was removed at the time of surgery; he recovered without complications and was discharged home on post-operative day 4. Pathological diagnosis of SNRCLA was made based on morphologic characteristics and immunohistochemistry (Table 1). Due to piecemeal resection via endoscopic approach, margin status was unclear. PET scan two months post-operatively suggested residual tumor along the nasal cavity and right orbit. The patient was referred for radiotherapy (RT) where he received 6000 cGy in 30 fractions with a boost of 600 cGy in three fractions to treat residual disease.

**Table 1:** Morphologic and immunochemistry results. Findings support the pathologic diagnosis of sinonasal renal cell-like adenocarcinoma.

Morphologic appearance	
Bland glandular cells forming tubules and follicles in a sheet-like pattern	
Follicular lumens contain serous secretions imparting a thyroid-like pattern	
Many cells contain moderate amounts of clear cytoplasm	
Lack of a basal layer (as demonstrated by negative immunostaining for p52)	
Immunostaining	
Positive	Negative
CK7, keratin 903, CAIX (weak), SDHB (weak), Ki-67 (variably low, focally approaches ~5%)	p63, ERG, TTF-1, BRAF, S-100, PAX-8, NKX3.1, CD30, Oct3/4, mammaglobin, CK20, CDX2, CD10, inhibin, chromogranin

After completion of RT, PET imaging at seven months post-op showed persistent disease that had a partial response to RT. Salvage surgery was pursued via combined open and endonasal excision where negative margins were eventually obtained. After salvage surgery, his treatment course was complicated by nasal wound healing complications. He required multiple reconstructive procedures including a skin graft and paramedian forehead flap reconstruction. He is currently without evidence of disease at 16 months after diagnosis.

## Discussion

SNRCLA is a rare tumor of the sinonasal tract that does not classify as any of the previously described sinonasal primary tumors [5]. SNRCLA was first described by Zur et al. in 2002 as a clear cell carcinoma that resembled RCC but without any evidence of a primary renal tumor [2]. Shortly after this description, Moh’d Hadi et al. published a case with a similar description and both of these were later classified as the newly described SNRCLA. Histologically, the lesions can appear similar to other clear cell neoplasms and the differential diagnosis should be wide. Clear cell neoplasms that must be ruled out include primary salivary lesions, primary non-salivary clear cell lesions, and metastatic lesions. Primary salivary clear cell lesions may initially appear similar, such as acinic cell carcinoma, clear cell mucoepidermoid carcinoma, hyalinizing clear cell carcinoma, clear cell oncocytoma, clear cell myoepithelial carcinoma, and epithelial myoepithelial carcinoma [6]. Additionally, primary non-salivary clear cell lesions also appear similar: squamous cell carcinoma with clear cell differentiation, endolymphatic sac tumors, and paragangliomas. Metastatic clear cell lesions to be ruled out include RCC, melanoma, clear cell sarcoma, and clear cell thyroid carcinoma [6]. Immunohistochemistry is used to distinguish SNRCLA from these lesions. In general, PAX8 and PAX2 are used to rule out RCC as they represent urogenital tumor origin and are not present in SNRCLA. RCC is also usually negative for CK7, CK20 and positive for vimentin, CD10, RCC antigen, and CA9 [4]. Positive staining for CK7 is characteristic of SNRCLA [4]. Since the description of SNRCLA in 2002, there have been multiple pathological studies on the disease, but information regarding the clinical behavior of the tumor remains sparse.

A literature review yielded 14 previously reported cases of SNRCLA, making ours the fifteenth case (Table 2) [2,3,6-12]. Cases that were not published in the English language or had a questionable pathologic diagnosis of SNRCLA were excluded from the review. Because this is a newly described disease, many case reports initially reviewed had a questionable diagnosis of SNRCLA and the diagnosis was reported as “clear cell neoplasms.” Although some literature reviews have subjectively assigned a diagnosis of SNRCLA to these historic reports, we chose to deliberately exclude cases where the clinical or pathological description did not strongly point to a diagnosis of SNRCLA. Therefore, our literature review and case series may slightly differ from others in the literature. In our review, patients were aged 22-89 years, six were male and nine were female. Additional patient characteristics were not consistently reported and hence further associations could not be made in this review. The most common presenting symptoms were epistaxis and nasal obstruction. Only one patient reported by Storck et al. did not undergo surgical resection as primary treatment and had primary RT with no recurrence at 24 months [11]. Otherwise, all other cases reported underwent primary surgical resection. Eight, including our case, underwent additional RT and none received systemic therapy. Wu et al. reported the only known case of local recurrence [9]. Their patient was treated with surgical excision alone and was found to have a recurrence 35 months after primary resection. Zhenwei et al. reported a patient who underwent surgical excision followed by adjuvant RT and at six months had “no disease progression" [10]. It is unclear based on the report whether this patient had a persistent disease or had no evidence of disease (NED) at follow-up. In the present case, we report persistent disease after surgical excision and RT eventually requiring salvage surgery. Although our patient is currently NED at 16 months, he did have multiple wound complications after salvage surgery attributed to poor wound healing in a radiated field. Otherwise, nine cases were NED at follow up and the remaining three cases reviewed were lost to follow-up/status not reported.

**Table 2:** Fifteen cases of SNRCLA reported in the literature. NR: not reported, LTF: lost to follow-up, NED: no evidence of disease. Status at follow-up is reported as stated in articles reviewed.

Case	Author	Age (years)	Sex	Location	Presenting symptoms	Surgery	Margin status	Radiation	Time to follow-up	Status at follow-up
1	Zur et al. [2]	50	F	Left nasal cavity	Epistaxis, headache, nasal/ocular pressure, epiphora	Craniofacial resection	NR	Adjuvant	NR	NR
2	Storck et al. [11]	36	F	Nasal NOS	NR	Surgical excision	NR	Adjuvant	48 mo	No recurrence
3	Storck et al. [11]	69	M	Nasopharynx	NR	None	NR	Primary definitive	24 mo	No recurrence
4	Shen et al. [12]	56	F	Nasal skull base	NR	Surgical excision	NR	Adjuvant	22 mo	No recurrence
5	Shen et al. [12]	89	F	Sinonasal	NR	Surgical excision	NR	No	4 mo	No recurrence
6	Shen et al. [12]	73	M	Nasal NOS	NR	Surgical excision	NR	Adjuvant	20 mo	No recurrence
7	Kim et al.[4]	63	M	Nasal NOS	Epistaxis, nasal obstruction, hyposmia	Craniofacial resection, bifrontal craniotomy combined endonasal endoscopic approach, en-bloc removal with dural resection	NR	Adjuvant	15 mo	No recurrence
8	Kubik et al.[6]	80	F	Nasal NOS	Asymptomatic	Combined endoscopic and open craniofacial resection: right transfrontal orbital approach with craniectemy by bicoronal incision, resection of dural and periorbital margins, reconstruction pericardial dural graft, and pericranial flap	Negative	No	NR	NR
9	Zhenwei et al. [10]	63	M	Left nasal cavity and choana	Epistaxis, nasal obstruction, headache	Surgical excision	NR	Adjuvant	6 mo	No disease progression
10	Wu et al.[9]	26	F	Nasal NOS	Epistaxis, nasal obstruction, and hyposmia x 1 year	Bicoronal incision with ligation of anterior ethmoid artery followed by endoscopic resection, reconstruction with pericranial and septal flaps	NR	No	24 mo	No recurrence
11	Wu et al. [9]	42	M	Nasal NOS	Epistaxis	Endoscopic resection	NR	No	35 mo	Recurrence
12	Brandwein-Gensler et al. [7]	56	F	Nasal skull base	NR	Resection	NR	Adjuvant	3 mo	NED
13	Moh'd Hadi et al.[3]	22	F	Right nasal cavity, posterior ethmoid	Epistaxis, nasal obstruction	Pre-op embolization, excision of the tumor via Weber-Ferguson incision with medial maxillectomy, ethmoidectomy	NR	No	4 years	NED
14	Negahban et al. [8]	52	F	Left maxillary and ethmoid sinuses	Left cheek mass, nasal discharge	Resection with hemimaxillectomy	NR	No	LTF	LTF
15	Present study	28	M	Bilateral ethmoid sinuses, skull base	Epistaxis, nasal obstruction, hyposmia, headache	Combined endonasal and open resection: bilateral ethmoidectomy and frontal sinusotomy, frontal sinus cranialization via bicoronal approach and pericranial flap	Positive	Adjuvant	16 mo	Persistent disease and re-excision at 7 mo, NED at 16 mo

## Conclusions

SNRCLA is a rare entity, with previous studies focusing on the pathologic characteristics instead of the clinical behavior of the disease. Our literature review supports that SNRCLA is a low-grade malignancy, with a low recurrence rate and no reported cases of metastases. Primary treatment with surgical excision is the mainstay of treatment; however, location along the skull base can lead to difficult surgical management. The role of RT has yet to be determined and further studies are needed to determine best treatment management.
